# Spatial assessment of forest soil carbon and climate-related value inside and outside protected areas in China

**DOI:** 10.1007/s10661-026-15630-5

**Published:** 2026-06-26

**Authors:** Yuxian Zhang, Guojie Wang, Pedro Cabral

**Affiliations:** 1 School of Remote Sensing and Geomatics Engineering, Nanjing University of Information Science & Technology, Nanjing, 210044 China; 2 NOVA Information Management School (NOVA IMS), Universidade Nova de Lisboa, Campus de Campolide, 1070-312 Lisbon, Portugal

**Keywords:** Soil organic carbon, Ecosystem services, Environmental assessment, Land-use stability, Carbon sequestration

## Abstract

Protected areas are widely used as spatial instruments for climate mitigation, yet their contribution to below‐ground carbon storage remains insufficiently quantified at national scales. This study assesses how forest soil organic carbon (SOC) and its climate-related value differ between protected and non-protected areas across China under historical and future climate conditions. Using field-based SOC observations and spatial machine‐learning models, we estimated SOC density at two soil depths (0–20 cm and 0–100 cm) from 2000 to 2100 under multiple Shared Socioeconomic Pathway scenarios. Spatially explicit SOC projections were generated using Random Forest models driven by climatic, vegetative, soil, and topographic variables, and SOC differences associated with protected-area status were evaluated through comparative analysis of forested regions inside and outside established reserves. The results reveal strong spatial differentiation in SOC trajectories, with stability or accumulation under low-emission pathways and widespread losses under high-emission scenarios, particularly in deeper soil layers. Forests within protected areas consistently exhibit higher SOC densities than unprotected forests, with mean differences of 45.5% in topsoil and 33.4% across the full soil profile. Extrapolation to protected areas established after 2000 indicates potentially substantial additional SOC stocks, corresponding to climate-related values ranging from tens to over one hundred billion USD under alternative carbon price assumptions. Although spatially independent validation indicates reduced predictive performance at fine scales, the model robustly captures broad climatic and edaphic gradients. Overall, this study provides a spatially explicit assessment of forest SOC dynamics to support conservation planning and climate-mitigation strategies.

## Introduction

Soil organic carbon (SOC) plays a critical role in the terrestrial carbon balance and climate-regulation system, storing nearly twice as much carbon as the atmosphere and acting as a major buffer against rising concentrations of CO₂ (J. Li et al., 2020; Qing et al., 2025). Forest ecosystems are especially important in this regard: although they occupy roughly one third of the Earth’s land surface, they contain more than 70% of global SOC stocks (Tian et al., 2016). Changes in forest SOC therefore have far-reaching implications not only for global carbon-climate feedbacks but also for soil fertility, nutrient cycling, and ecosystem productivity (Chen et al., 2023). Processes such as erosion and land degradation can accelerate SOC losses, further amplifying atmospheric CO₂ emissions (Rao et al., 2023). As countries pursue long-term climate-neutrality strategies, enhancing carbon storage in terrestrial ecosystems, particularly forests, has become a key governance priority (Griscom et al., 2017; Tian et al., 2023). Global assessments increasingly emphasize land-based climate solutions, including soil-carbon protection, as essential components of viable mitigation pathways (IPBES, 2019).

Despite this recognition, projecting future SOC dynamics remains deeply uncertain. SOC responds nonlinearly to interacting climatic, vegetative, edaphic, and disturbance processes, making its trajectory highly sensitive to spatial context. Although process-based and Earth system models aim to capture these interactions, they often diverge substantially in their predictions of SOC change, reflecting persistent uncertainty in carbon–climate feedbacks and limited parameterization of fine-scale heterogeneity (Pierson et al., 2022; Shi et al., 2024; Zhao et al., 2021). Reducing these uncertainties requires approaches that explicitly represent spatial variability and integrate both biophysical conditions and management or governance contexts. Evidence from Northeast China, where black soils have experienced pronounced SOC declines following land-use conversion and agricultural intensification, illustrates that governance and land management can modulate, and in some cases override, climatic influences on soil-carbon stability (Li et al., 2025a, 2025b). At the global scale, assessments similarly emphasize that conservation outcomes depend strongly on governance quality, institutional capacity, and local implementation contexts, all of which shape ecological responses to climate change (Eklund & Cabeza, 2017; IPBES, 2019).

Recent advances in machine‐learning techniques have substantially strengthened digital soil mapping (DSM), which seeks to predict the spatial distribution of soil properties by modeling relationships between field observations and environmental covariates. DSM is commonly conceptualized through the scorpan framework, in which soil properties are expressed as functions of soil-forming factors, including climate (c), organisms (o), relief (r), parent material (p), age (a), and spatial position (n). Within this framework, machine‐learning algorithms provide flexible tools to model nonlinear and spatially variable relationships between SOC and environmental gradients such as climate, vegetation indices, and topography (Bócoli et al., 2025). These data-driven approaches have proven effective for large-area SOC mapping and spatial diagnosis, often complementing or extending traditional statistical and process-based methods (Barrena-González et al., 2023; Morais et al., 2023; Taghizadeh-Mehrjardi et al., 2020). Random Forest models, in particular, have been widely adopted to quantify SOC across heterogeneous landscapes while limiting overfitting and accommodating complex interactions among predictors (Nwaogu et al., 2024; van der Westhuizen et al., 2023). Nevertheless, most SOC modelling remains retrospective or static, with limited attention to future climate trajectories or to the role of spatial governance structures. As a result, it remains unclear where, and under which climatic conditions, protected areas generate distinct soil-carbon outcomes relative to surrounding, non-protected landscapes (Eklund & Cabeza, 2017; Watson et al., 2018).

China provides a particularly relevant context for addressing this gap. The country spans broad climatic and edaphic gradients and contains forest ecosystems that are highly sensitive to ongoing warming and drying trends (Li et al., 2025a, 2025b). At the same time, China has implemented one of the world’s largest expansions of protected areas, now covering more than 18% of its national territory (Fan et al., 2023). These protected areas are widely assumed to safeguard carbon stocks by limiting deforestation, promoting ecological restoration, and regulating land-use pressures. However, empirical evidence of their effectiveness in conserving soil carbon, especially from a spatially explicit perspective that distinguishes protected and non-protected territories, remains scarce. Existing national assessments tend to emphasize total carbon sequestration or vegetation-based indicators (Tian et al., 2023), leaving below-ground SOC processes comparatively underexamined. Moreover, it remains uncertain whether protected-area status confers greater resistance or stability of SOC under future climate change across China’s diverse forest and climate zones, despite the clear relevance of this question for spatial planning, conservation prioritization, and climate adaptation.

Beyond its biophysical role, SOC also represents a spatially relevant climate-mitigation asset. Stored soil carbon can be expressed in CO₂-equivalent terms using standard molecular ratios, allowing indicative valuation based on social cost of carbon estimates, which commonly range between 30 and 150 USD t⁻1 CO₂ (Bartkowski et al., 2020; Hungate et al., 2017; Keesstra et al., 2016). While such valuation does not represent market behavior or policy incentives directly, it provides a useful metric for comparing the climate-related value of carbon retained across different territories. Systematic spatial valuation of forest SOC in China, particularly in relation to protected and non-protected forests, remains limited, yet it has the potential to support place-based decision-making and to clarify the broader societal benefits associated with conservation governance.

In this study, we integrate machine‐learning-based SOC mapping, long-term climate projections, and empirical SOC observations to evaluate how protected-area governance shapes forest soil-carbon dynamics across China. We estimate SOC density at two soil depths (0–20 cm and 0–100 cm) from 2000 to 2100 under multiple Shared Socioeconomic Pathway (SSP) scenarios using models trained on climatic, vegetation, topographic, and soil predictors. To avoid circularity between modelled SOC patterns and protection status, protected-area effects are assessed using independent SOC sampling points. We then extrapolate these spatial differences to protected areas established after 2000 and translate retained SOC stocks into CO₂-equivalent values to derive indicative climate-related economic value. Specifically, this study aims to (i) quantify historical and projected spatial patterns of forest SOC across soil depths, (ii) assess whether protected forests exhibit consistently higher SOC densities than surrounding non-protected forests, (iii) evaluate how protected-area effects on SOC vary under future climate scenarios, and (iv) estimate the spatial distribution of climate-related value associated with SOC retention across protection regimes.

By explicitly linking spatial governance, climate forcing, and below-ground carbon dynamics, this study provides an applied geographic perspective on where and under which conditions protected areas deliver disproportionate climate-mitigation value. The findings offer policy-relevant insights for spatial prioritization and conservation planning, highlighting soil carbon as a critical yet often overlooked component of land-use and climate governance.

## Materials and methods

### Study area

The study area is China, located in East Asia and spanning from 73° 33′ E to 135° 05′ E and 18° 05′ N to 53° 33′ N, covering approximately 9.6 million km^2^. China exhibits strong topographic gradients that influence climatic and ecological patterns.

**Fig. 1:**
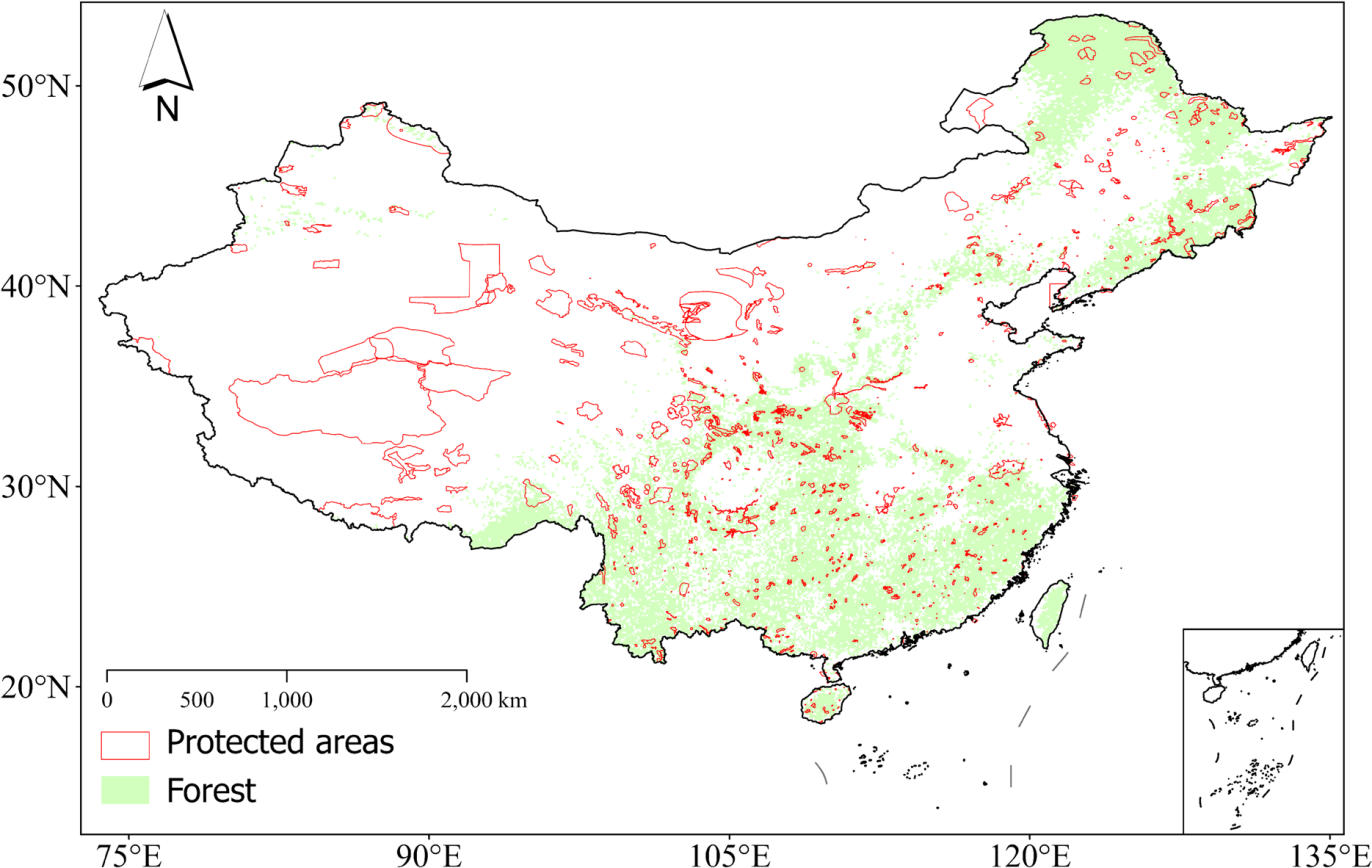
Spatial distribution of protected areas and forest cover in 2010

A total of 1028 protected areas vector elements were downloaded from the China Nature Reserve Specimen Resource Sharing Platform. Figure 1 shows the distribution of protected areas and the forest distribution in 2010. Protected areas are predominantly concentrated in the western region, where they are extensive and dense, moderately distributed in the southern region, and sparse with smaller coverage in the eastern region. Forest cover map was extracted based on land cover data. After removing protected areas established after 2000, 743 protected areas vector elements remained. Overlay analysis revealed that in 2000, the total forest area in China was 2,398,350.00 km^2^, and the forest area of protected areas was 94,729.76 km^2^, accounting for 3.97%.

### Soil organic carbon observations

SOC observations for two standard depth intervals (0–20 cm and 0–100 cm) were extracted from the forest ecosystem subset of the China Terrestrial Ecosystem Carbon Density Dataset for the 2010 s (Xu et al., 2020), distributed through the National Ecosystem Science Data Center (http://www.nesdc.org.cn).

**Fig. 2:**
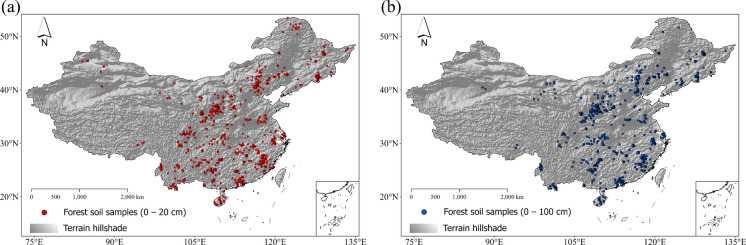
Forest samples distribution at 0–20 cm (**a**) and 0–100 cm (**b**) depths. The background hillshade represents terrain relief to provide geographic context for sample locations

After data screening, a total of 1664 forest soil samples were retained for the 0–20 cm layer and 1248 samples for the 0–100 cm layer (Fig. 2). These samples cover a wide range of forest ecosystems across China and provide a robust basis for modeling the spatial distribution of forest soil organic carbon.

The selection of the 0–20 cm and 0–100 cm depth intervals reflects both dataset consistency and their relevance for large-scale carbon assessments. The 0–20 cm layer represents the biologically active topsoil, while the 0–100 cm interval corresponds to the cumulative soil carbon stock within the full rooting zone, which is commonly used in national and global carbon inventories. Using these standardized layers avoids additional uncertainty associated with deriving intermediate layers (e.g., 20–100 cm) through subtraction, which could introduce error propagation. This depth configuration therefore supports both surface sensitivity analysis and full-profile carbon stock evaluation.

### Environmental predictors and spatial data

This study follows the DSM framework, in which SOC is modeled as a function of environmental covariates derived from climate, vegetation, and topographic data. A suite of environmental drivers was assembled to support SOC modeling under both historical and future conditions.

The Global Land Surface Satellite (GLASS) product was employed to obtain leaf area index (LAI) data. LAI reflects vegetation density and photosynthetic activity, which are closely linked to organic matter input into soils. This dataset (Ma & Liang, 2022), derived from MODIS reflectance using an advanced general regression neural network algorithm, provides long-term spatially continuous observations at 250–500 m resolution from 2000 to the present, enabling large-scale vegetation monitoring.

According to the Köppen climate classification (Kottek et al., 2006), the country can be divided into six major climatic zones: humid, cold semi-humid, temperate semi-humid, Qinghai-Tibet Plateau, arid, and semi-arid.

Monthly temperature and precipitation data at 1 km spatial resolution were obtained from the National Tibetan Plateau Data Center (Peng, 2019, 2020). These two climate datasets are China-specific datasets generated by fusing CRU global climate data with WorldClim high-resolution data using the Delta spatial downscaling scheme and have been optimized for China’s topographic features. Temperature and moisture regimes control organic matter decomposition rates and microbial activity, making them critical drivers of SOC dynamics (Liu et al., 2016). Annual mean temperature and precipitation were calculated from the downscaled monthly records. Digital elevation model (DEM) data (CGIAR-CSI, 2013) were also obtained through the same data portal at a spatial resolution of 0.00833°. Based on the DEM, terrain attributes including slope, aspect, and the Topographic Wetness Index (TWI) were calculated using ArcGIS Pro. These topographic factors influence water retention, soil erosion, and microclimate, all of which affect SOC distribution (Xin et al., 2016).

Soil type and soil texture data were sourced from the Data Center for Resources and Environmental Sciences, Chinese Academy of Sciences (https://www.resdc.cn/). The datasets were derived from the “People’s Republic of China 1:1,000,000 Soil Map” compiled by the National Soil Survey Office in 1995. Soil type information at the soil-order level (12 orders nationwide) was used, and soil texture was represented by quantitative fractions of sand, silt, and clay. Soil properties determine SOC stabilization, nutrient availability, and carbon storage capacity (Meersmans et al., 2008).

A 30 m land-cover dataset (Yang & Huang, 2021) was used to derive the Forest Fragmentation Index (FFI). Forest pixels were assigned a value of 1, with other land-cover classes assigned 0. FFI integrates three normalized metrics, i.e., edge density (ED), patch density (PD), and mean patch area (MPA), into a dimensionless indicator ranging from 0 to 1, with higher values indicating stronger fragmentation (Ma et al., 2023). Forest fragmentation affects litter input, microclimate, and soil disturbance, influencing local SOC dynamics. Calculations were performed in Python.

Future climate predictors (mean annual temperature and precipitation) were taken from the statistically downscaled CMIP6 projections provided by the WorldClim (Fick & Hijmans, 2017). These projections are generated from multiple global climate models (GCMs) under the SSP scenarios developed in CMIP6 (Eyring et al., 2016), which integrate assumptions of socioeconomic development, greenhouse gas emissions, and land-use trajectories. In this study, we used ensemble averages based on 13 GCMs for each SSP and time period to reduce model-specific biases. It is noteworthy that the current climate predictors employed in this study were derived from the TPDC 2000–2020 dataset, which was generated through the Delta spatial downscaling approach by fusing CRU global climate data with the WorldClim high-resolution climate surfaces (Peng, 2019, 2020). This methodological linkage ensures that our baseline climate data shares a common data lineage with the WorldClim CMIP6 projections, as both incorporate WorldClim’s high-resolution spatial information. Such inherent compatibility between the current and future climate datasets enhances the robustness of our climate change impact assessments by reducing potential discrepancies arising from fundamentally different data sources.

Future land-cover maps for 2020–2100 were obtained from a 1-km CMIP6-driven projection dataset (Zhang et al., 2023). Future LAI data were sourced from a recently developed dataset integrating CMIP6 climate projections with biome-specific vegetation models to generate continuous global LAI series for the twenty-first century (Li et al., 2024).

Due to the absence of annual forest-type datasets, forest categories were harmonized across time by aligning land-cover-based forest patches with the 1:1,000,000 basic vegetation map using a nearest-neighbor approach. This method assumes gradual rather than abrupt transitions among forest types and preserves temporal consistency in classification.

The period 2000–2020 was defined as the historical baseline, whereas 2021–2100 was considered the future period. The future interval was further divided into four 20-year sub-periods (2021–2040, 2041–2060, 2061–2080, and 2081–2100) to facilitate trend analysis. Table 1 summarizes the characteristics of the datasets used in this study.

**Table 1:** Datasets considered and used in this study

Dataset	Abbreviations	Spatial resolution	Units	Temporal coverage	Source
Leaf Area Index	LAI	0.05°	m^2^ m⁻^2^	1982–2022	(Li et al., 2024)
Temperature	TEMP	0.00833°	0.1 ℃	1901–2023	(Peng, 2019)
Precipitation	PRE	0.00833°	0.1 mm	1901–2023	(Peng, 2020)
Digital elevation Model	DEM	0.00833°		2000	(CGIAR-CSI, 2013)
Soil type	ST	1 km		1995	https://www.resdc.cn/
Land cover		30 m		1985–2024	(Yang & Huang, 2021)
Climate zone	CZ				(Kottek et al., 2006)
Soil texture		1 km	%	1995	https://www.resdc.cn/
Future LAI		0.05°	m^2^ m⁻^2^	1983–2100	(Li et al., 2024)
Future climate		0.00833°		2021–2100	https://www.worldclim.org
Future land-cover		1 km		2020–2100	(Zhang et al., 2023)

### Methods

#### Spatial preprocessing and predictor screening

All environmental drivers were projected to the WGS 1984 geographic coordinate system and resampled to a spatial resolution of 0.0833°. This resolution represents a compromise between preserving broad spatial gradients in climate, vegetation, and soil properties and ensuring computational consistency at the national scale and is appropriate for assessing spatial governance patterns associated with protected areas across China. Continuous variables were resampled using bilinear interpolation, whereas categorical variables were processed using nearest-neighbor resampling to preserve thematic accuracy. Prior to model development, multicollinearity among predictor variables was examined using the variance inflation factor (VIF) (An et al., 2024), and variables with a VIF greater than 10 were removed. Variables exhibiting excessive collinearity were removed to minimize redundancy and improve model robustness.

#### Spatial modeling and algorithm selection

After confirming acceptable collinearity levels, the dataset was randomly partitioned into training (80%) and validation (20%) subsets, and five-fold cross-validation was further applied on the training set to optimize model performance and assess its robustness. In addition to standard cross-validation, we implemented spatially blocked cross-validation to reduce potential optimistic bias arising from spatial autocorrelation among sampling points (Pohjankukka et al., 2017; Roberts et al., 2017). A regular spatial grid of 1° × 1° was used to partition the study area into 243 spatial blocks, which were subsequently employed in a five-fold spatial block cross-validation. Each block contained approximately 6–7 samples, ensuring spatial independence while avoiding excessive sparseness. Folds were generated by withholding entire blocks at each iteration, and model performance was summarized as mean ± standard deviation across folds, providing a more conservative estimate of spatial generalization ability.

Three machine-learning algorithms, i.e., Random Forest (RF) (Leo, 2001), Decision Tree (DT) (Loh, 2011), and Extreme Gradient Boosting (XGBoost (Chen & Guestrin, 2016), were evaluated, and the algorithm demonstrating the most stable and accurate performance was selected to generate spatial predictions of SOC. The purpose of this comparison was to ensure robust spatial estimation rather than to optimize algorithmic performance.

#### Ensemble modeling and uncertainty quantification

To quantify model uncertainty arising from stochastic learning processes, an ensemble modeling framework was implemented (Yang et al., 2023). Ten independent models were trained using identical hyperparameters but distinct random seeds, allowing variation associated with bootstrap sampling and random feature selection. Ensemble SOC predictions were calculated as the mean of the ten simulations for each grid cell and time period, while the standard deviation among ensemble members was interpreted as model-related uncertainty.

#### Climate scenario integration and future SOC projection

Future SOC projections further incorporated climate projection uncertainty by using outputs from 13 global climate models (GCMs) under the CMIP6 framework. SOC predictions were generated separately for each GCM using the trained machine‐learning model, and final future estimates were obtained by averaging across GCM-specific predictions. For each scenario and soil depth, annual SOC predictions were aggregated to multi-year means corresponding to the defined historical and future sub-periods to facilitate temporal comparison. 

#### Assessment of protected-area associations with soil organic carbon

To evaluate spatial differences in SOC associated with protected-area status, SOC density was statistically compared between forested regions inside and outside nature reserves. Because most SOC measurements in China were conducted after 2000, reserves established in or after 2000 were excluded to avoid temporal ambiguity regarding protection status. Differences in SOC distributions were assessed using the non-parametric Mann–Whitney *U* test. To quantify uncertainty in mean SOC differences, bootstrap resampling with replacement was repeated 5000 times to derive 95% confidence intervals, an approach suitable for datasets with unequal sample sizes and non-normal distributions. These comparisons reflect long-term spatial associations between protection status and soil carbon rather than causal effects of protected-area designation.

#### Extrapolation and climate-related economic valuation

To extend conservation implications beyond historically established reserves, the per-unit-area SOC benefit estimated from reserves established before 2000 was extrapolated to forest areas newly incorporated into protected areas after 2000. This extrapolation assumes that forests added to protected areas after 2000 would experience SOC responses comparable to those observed in long-established reserves under similar protection regimes. Changes in SOC stocks were converted to CO₂-equivalent units using the molecular mass ratio of CO₂ to C. To estimate indicative climate-related economic values for spatial comparison, five carbon price scenarios (10, 30, 50, 70, and 100 USD t⁻1 CO₂) were applied consistently to both historically established and newly designated protected forest areas.

The extrapolated total potential SOC gain is calculated as follows:

1 $$\triangle SOC_{pro}=\triangle SOC_{pre-2000}\times A_{new}$$

$\triangle SOC_{pre-2000}$ represents the per-unit-area difference in SOC density between forests inside and outside protected areas established before 2000, whereas $A_{new}$ denotes the forest area newly incorporated into protected areas after 2000.

The extrapolated potential economic SOC value is calculated as follows:

2 $$V=\triangle SOC_{pro}\times\frac{44}{12}\times P_{CO_2}$$

where $P_{CO_2}$ refers to the carbon price $\left(USD\;t^{-1}CO_2\right)$

## Results

### Model performance and spatial reliability

Under the standard 80/20 random split and five-fold cross-validation, XGBoost consistently achieved the highest explained variance and the lowest prediction errors across both soil depths, followed closely by Random Forest (Table 2). However, model performance declined under spatial block five-fold cross-validation, particularly for the 0–100 cm layer, reflecting the greater challenge of spatial extrapolation. In this framework, Random Forest outperformed all other models at both depths.

**Table 2:** Performance of different models in predicting SOC density at two soil depths, evaluated using the coefficient of determination (*R*^2^), mean squared error (*MSE*), and mean absolute error (*MAE*). Test set results are presented first, followed by standard and spatially blocked fivefold cross-validation (*CV*) results, reported as mean ± standard deviation (*SD*)

Soil depth	Model	*R*^2^	RMSE	MAE	Spatial block CV *R*^2^ (mean ± SD)	Spatial block CV RMSE (mean ± SD)	Spatial block CV MAE (mean ± SD)
0–20 cm	XGBoost	0.536 (0.504 ± 0.057)	1.83 (2.03 ± 0.10)	1.30 (1.44 ± 0.04)	0.360 ± 0.062	2.26 ± 0.08	1.73 ± 0.06
	Decision Tree	0.404 (0.398 ± 0.108)	2.08 (2.46 ± 0.16)	1.47(1.66 ± 0.10)	0.154 ± 0.111	2.59 ± 0.12	2.01 ± 0.10
	Random Forest	0.529 (0.517 ± 0.061)	1.85 (2.0 ± 0.11)	1.30 (1.44 ± 0.05)	0.371 ± 0.065	2.24 ± 0.10	1.73 ± 0.07
0–100 cm	XGBoost	0.363 (0.463 ± 0.094)	4.91 (4.58 ± 0.46)	3.42 (3.16 ± 0.23)	0.195 ± 0.033	6.82 ± 0.84	5.12 ± 0.48
	Decision Tree	0.164(0.176 ± 0.107)	5.62(5.66 ± 0.27)	3.80(3.71 ± 0.17)	−0.235 ± 0.106	6.91 ± 1.20	5.00 ± 0.74
	Random Forest	0.326 (0.454 ± 0.108)	5.05 (4.61 ± 0.52)	3.49 (3.22 ± 0.25)	0.214 ± 0.034	5.45 ± 0.64	4.13 ± 0.35

Although XGBoost yielded slightly higher accuracy under conventional random cross-validation, Random Forest demonstrated superior performance under spatial cross-validation, indicating stronger spatial generalization and reduced susceptibility to overfitting spatial autocorrelation. Given the small performance differences between the two models under random validation, Random Forest was ultimately selected for spatial prediction of soil organic carbon and subsequent analyses.

### Spatial patterns of SOC change under future climate scenarios

By the end of the twenty-first century, the trend of forest soil organic carbon density relative to historical baselines showed a clear scenario-dependent pattern and significant vertical differences among forest regions in China. Under all three scenarios, topsoil SOCD was projected to decline in most forest regions, with the extent and magnitude of the decline gradually increasing from SSP1-2.6 to SSP5-8.5. Under the low-emission scenario SSP1-2.6, the decline in topsoil SOCD was relatively small and spatially uneven; significant declines mainly occurred in South and Southeast China, while parts of Northeast China showed a near-neutral or even slightly increasing trend. In contrast, under SSP2-4.5, and especially SSP5-8.5, the decline in SOCD was more widespread and significant, with marked decreases observed in South, Central, and Eastern forest regions. The high-emission scenario (SSP5-8.5) resulted in the most severe SOCD loss in the 0–20 cm soil layer.

The 0–100 cm soil profile exhibited a generally consistent pattern but with significant vertical differences. Compared to the surface soil, SOCD losses in deeper soils were generally smaller under SSP1-2.6 scenarios, with even slight increases or minimal changes observed in parts of Northeast and Northern China. However, under SSP2-4.5 and SSP5-8.5 scenarios, the negative changes extended more broadly to deeper soil layers, particularly in Southern China, indicating that subsoil carbon is equally vulnerable under medium- to high-emission pathways. Overall, SOCD losses in both surface and deep soils increased significantly with increasing radiative forcing levels.

**Fig. 3:**
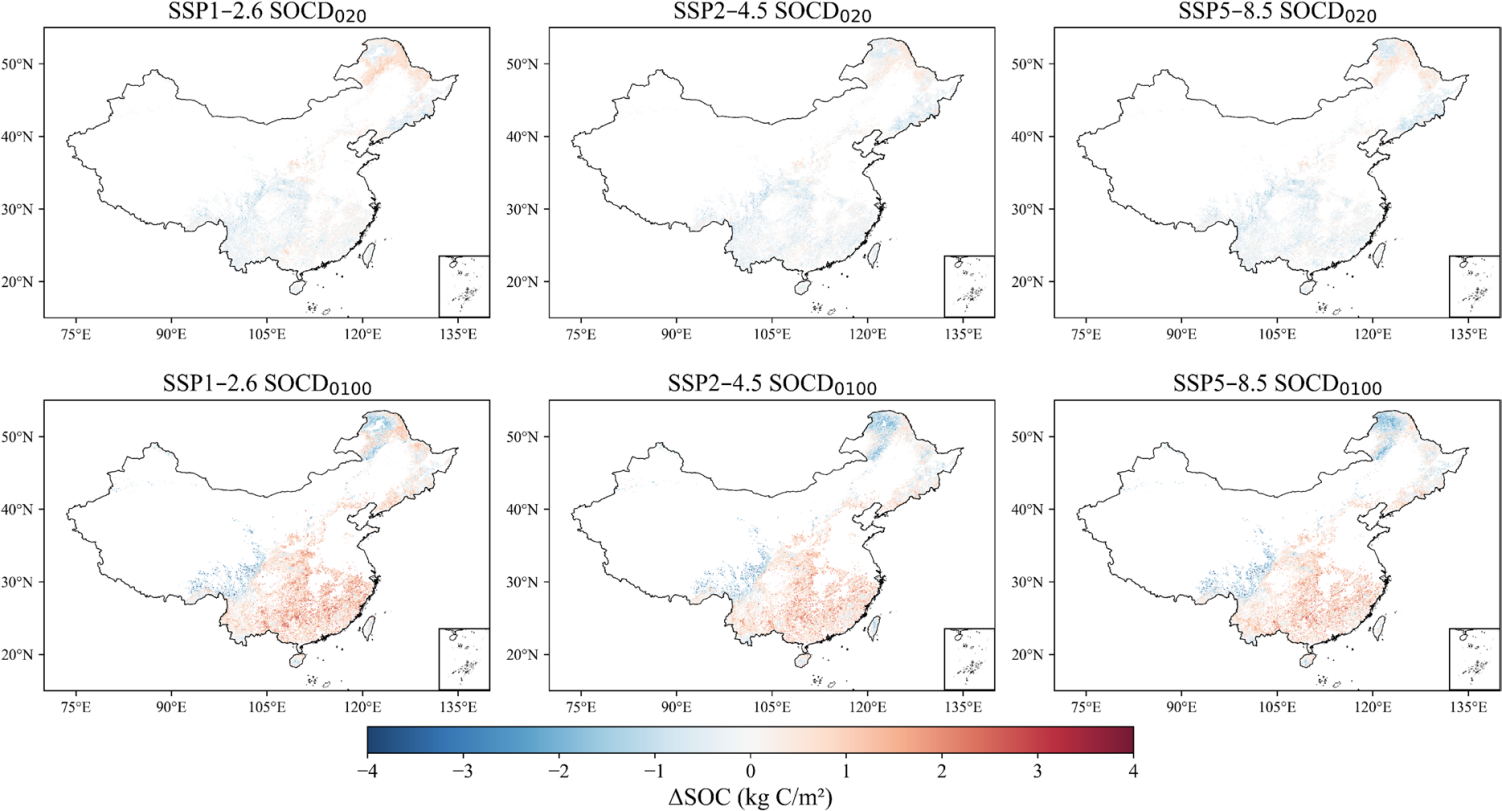
Projected changes in soil organic carbon (SOC) density relative to the historical period of 2000–2020 by the end of the twenty-first century under three soil organic carbon (SOC) scenarios (SSP1-2.6, SSP2-4.5, and SSP5-8.5). The first row, a–c, represents the SOC change in the topsoil (0–20 cm), and the second row, d–f, represents the SOC change in the entire soil profile (0–100 cm)

These spatial patterns highlight the high sensitivity of Chinese forest SOCD to climate. Southern forests appear particularly vulnerable to carbon loss, likely due to accelerated decomposition caused by rising temperatures and potential changes in precipitation patterns. In contrast, Northeastern forests exhibited greater resilience, especially under low-emission scenarios. The study results indicate that proactive mitigation measures consistent with SSP1-2.6 can significantly reduce the risk of soil carbon loss, while sustained high emissions (SSP5-8.5) will lead to a large-scale and more severe decline in SOCD in China’s forest ecosystems by 2100 (Fig. 3).

### Temporal evolution of SOC stocks and associated economic implications

**Fig. 4:**
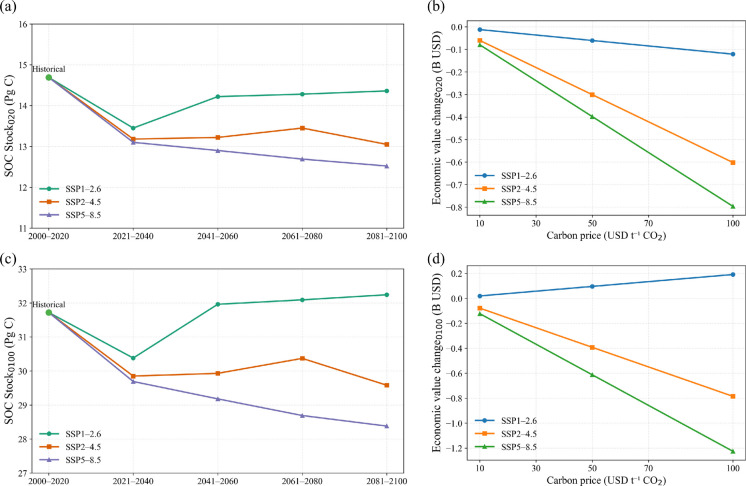
Changes in SOC stocks over time and corresponding climate-related economic values by the end of the century relative to 2000–2020. Panels (**a**) and (**c**) show SOC stock changes in the 0–20 cm and 0–100 cm layers, respectively, while panels (**b**) and (**d**) show associated economic values

Temporal trajectories of forest SOC stocks reveal pronounced differences among climate scenarios and soil depths (Fig. 4a, c). In the 0–20 cm surface layer, SOC stocks decline markedly from the historical baseline (2000–2020) under all scenarios during the early-to-mid twenty-first century. Under the low-emission SSP1-2.6 pathway, surface SOC stocks recover after the initial decline and increases steadily, reaching the highest end-of-century value among the scenarios, though still slightly below the historical level. Under SSP2-4.5, SOC shows only limited recovery after the initial decline, peaking slightly in the mid-century before decreasing again during 2081–2100. In contrast, SSP5-8.5 exhibits a continuous downward trend after 2040, resulting in the lowest SOC stock by the late century. Overall, the surface SOC stock exhibits a decreasing trend under all scenarios by the end of this century, consistent with the findings reported by Song et al., (2025). In the 0–100 cm soil layer, under the SSP1-2.6 scenario, deep soil organic carbon storage showed a declining trend from 2021 to 2040, followed by a continuous recovery and growth, with organic carbon storage exceeding historical levels by the end of this century; while under the SSP2-4.5 and SSP5-8.5 scenarios, it continued to decline. These results indicate that deep soil carbon is more susceptible to the effects of long-term climate warming, especially under high-emission scenarios.

The economic consequences closely mirror these biophysical changes (Fig. 4b, d). Relative to the historical baseline, all future scenarios result in economic losses except for the 0–100 cm layer under SSP1-2.6, with losses increasing almost linearly as carbon prices rise. Under SSP1-2.6, economic losses remain comparatively small, reflecting the relatively high retention of forest soil organic carbon (SOC). At a carbon price of US$100 t⁻^1^ CO₂, the estimated economic loss is approximately 0.8 billion USD for the 0–20 cm layer and 1.2 billion USD for the 0–100 cm layer. Losses increase substantially under SSP2-4.5 and are greatest under SSP5-8.5. These results indicate that low-emission pathways can markedly reduce SOC losses and their associated economic costs, whereas high-emission scenarios are likely to cause substantial declines in both carbon storage and ecosystem service value.

### Forest-type differentiation and protected-area effects on soil organic carbon

**Fig. 5:**
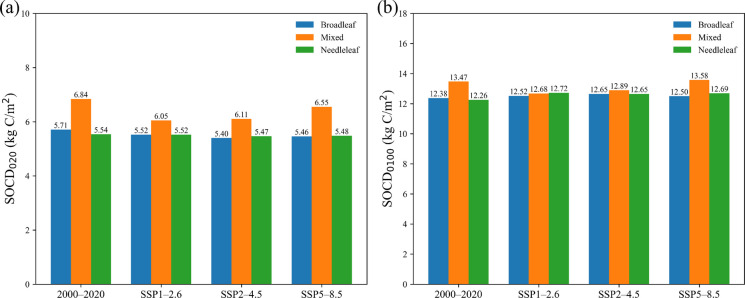
Changes in SOCD by forest type at the end of the century relative to 2000-2020 for the (**a**) 0–20 cm and (**b**) 0–100 cm layers

Forest SOCD showed pronounced differences among forest types and soil depths (Fig. 5). In the 0–20 cm layer, mixed forests consistently exhibited the highest SOCD across all periods, followed by broadleaf forests, whereas needleleaf forests had the lowest values. During 2000–2020, SOCD in mixed forests reached 6.84 kg C m⁻2, compared with 5.71 kg C m⁻2 in broadleaf forests and 5.54 kg C m⁻2 in needleleaf forests. Under future climate scenarios, surface SOCD declined in all forest types, with the largest reductions occurring in mixed forests and relatively minor changes in needleleaf forests.

In the 0–100 cm layer, mixed forests also maintained the highest SOCD, while broadleaf and needleleaf forests exhibited similar values. Unlike the surface layer, deep-soil SOCD remained comparatively stable under future climate change, with only slight fluctuations among scenarios. Mixed forests showed a modest increase under SSP5-8.5, although the magnitude of this change was relatively small and may reflect regional differences in productivity–decomposition balance and forest distribution under climate change (Davidson & Janssens, 2006).

**Fig. 6:**
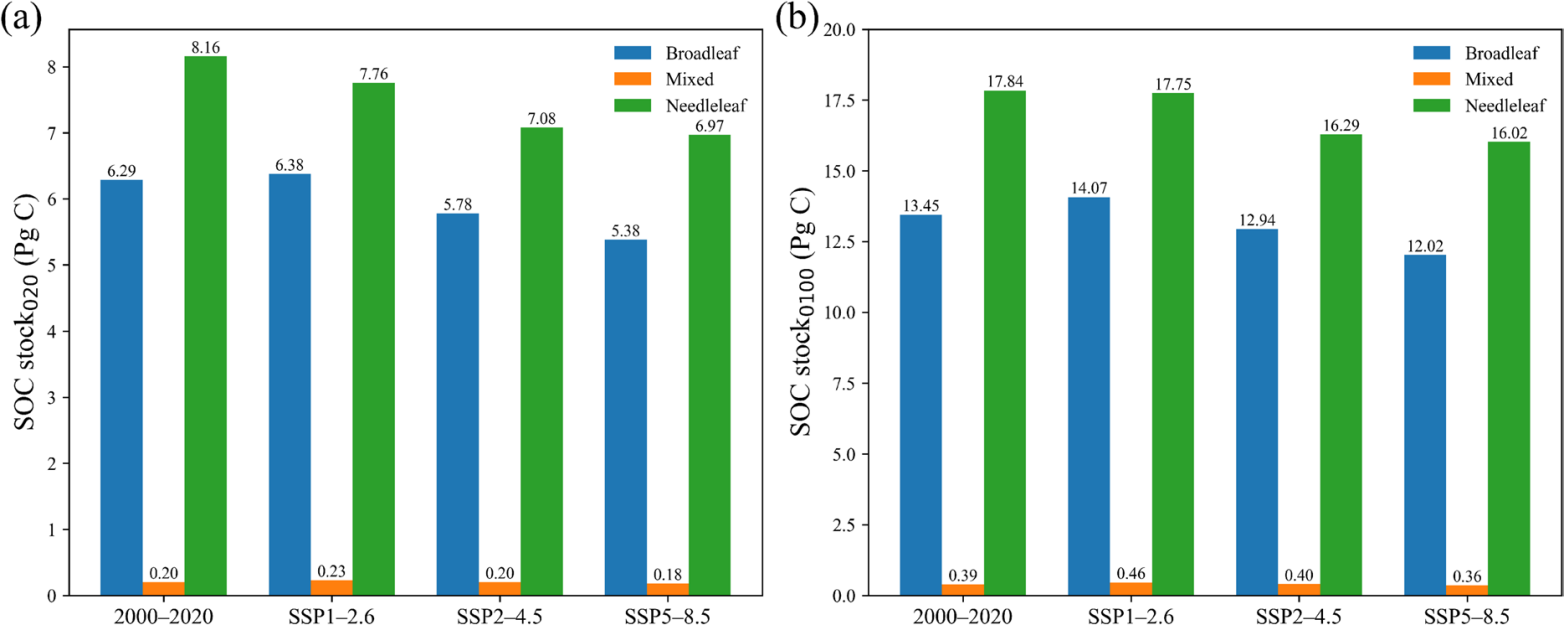
Changes in SOC stocks by forest type at the end of the century relative to 2000-2020 for the (**a**) 0–20 cm and (**b**) 0–100 cm layers.

Forest SOC stocks varied markedly among forest types and soil depths (Fig. 6). Needleleaf forests consistently stored the largest SOC stocks, followed by broadleaf forests, whereas mixed forests contributed only a negligible fraction. Although mixed forests exhibit the highest SOCD, their total SOC stocks are the lowest because they occupy a much smaller area than broadleaf and needleleaf forests. During 2000–2020, needleleaf forests accounted for the largest share of total forest SOC in both the 0–20 cm and 0–100 cm layers, substantially exceeding broadleaf and mixed forests.

Under future climate scenarios, SOC stocks generally declined across all forest types, although the magnitude of change differed considerably. Needleleaf forests exhibited the largest absolute decreases, whereas mixed forests remained relatively stable. Broadleaf forests showed moderate declines, despite a slight increase under SSP1-2.6, particularly in the 0–100 cm layer. Overall, needleleaf forests are projected to remain the dominant SOC reservoir in China’s forests and are likely to account for most future SOC losses under climate warming.

Under future climate scenarios, SOC trajectories diverge systematically among forest types and emission pathways. Coniferous and broadleaf forests both experience progressive SOC losses from low- to high-emission scenarios, with the strongest declines occurring under SSP5-8.5. In contrast, mixed forests exhibit comparatively smaller absolute changes, consistent with their broader climatic distribution and lower baseline SOC stocks. Across all forest types, SOC losses are disproportionately concentrated in the 0–100 cm soil profile, indicating that deeper soil carbon pools are more sensitive to long-term warming than surface soils (Fig. 5).

Beyond forest-type differences, protected-area status is associated with consistent and statistically significant SOC contrasts across soil depths (Table 3). Forests located within protected areas exhibit higher SOC densities than unprotected forests in both the 0–20 cm and 0–100 cm layers (*p* < 0.001). Mean SOC density inside protected areas is 45.5% higher in topsoil and 33.4% higher in the full soil profile. Effect sizes are moderate (Cliff’s Δ = 0.27–0.38), indicating persistent territorial differentiation rather than marginal differences.

**Table 3:** Results of the Mann–Whitney *U* test comparing SOC density between protected and unprotected forests

Soil depth	*Z*	*P*	*r*	Cliff’s Δ	Mean SOCD (inside)	Mean SOCD (outside)	% Difference
0–20 cm	8.90	< 0.001	0.22	0.38	6.40	4.41	45.5
0–100 cm	4.72	< 0.001	0.13	0.27	13.19	9.99	33.4

 Bootstrap analysis confirms the robustness of these contrasts, with estimated SOC enhancements of 2.0 kg m⁻² (95% CI: 1.56-2.45) in the 0-20 cm layer and 3.35 kg m⁻² (95% CI: 1.92-4.83) in the 0-100 cm profile (Table 4). When extrapolated to forest areas incorporated into protected areas after 2000, these per-unit-area differences correspond to additional SOC stocks of approximately 0.28 Pg C in topsoil and 0.47 Pg C across the full soil profile.

**Table 4:** Bootstrap-derived SOC differences associated with protected forests and the corresponding national-scale SOC stock increases estimated through area extrapolation

Soil depth	ΔSOC (kg m^−2^)	ΔSOC CI lower	ΔSOC CI upper	Added SOC stock (Pg C)	Stock CI lower (Pg C)	Stock CI upper (Pg C)
0–20 cm	2.00	1.56	2.45	0.28	0.22	0.35
0–100 cm	3.35	1.92	4.83	0.47	0.2	0.68

**Fig. 7:**
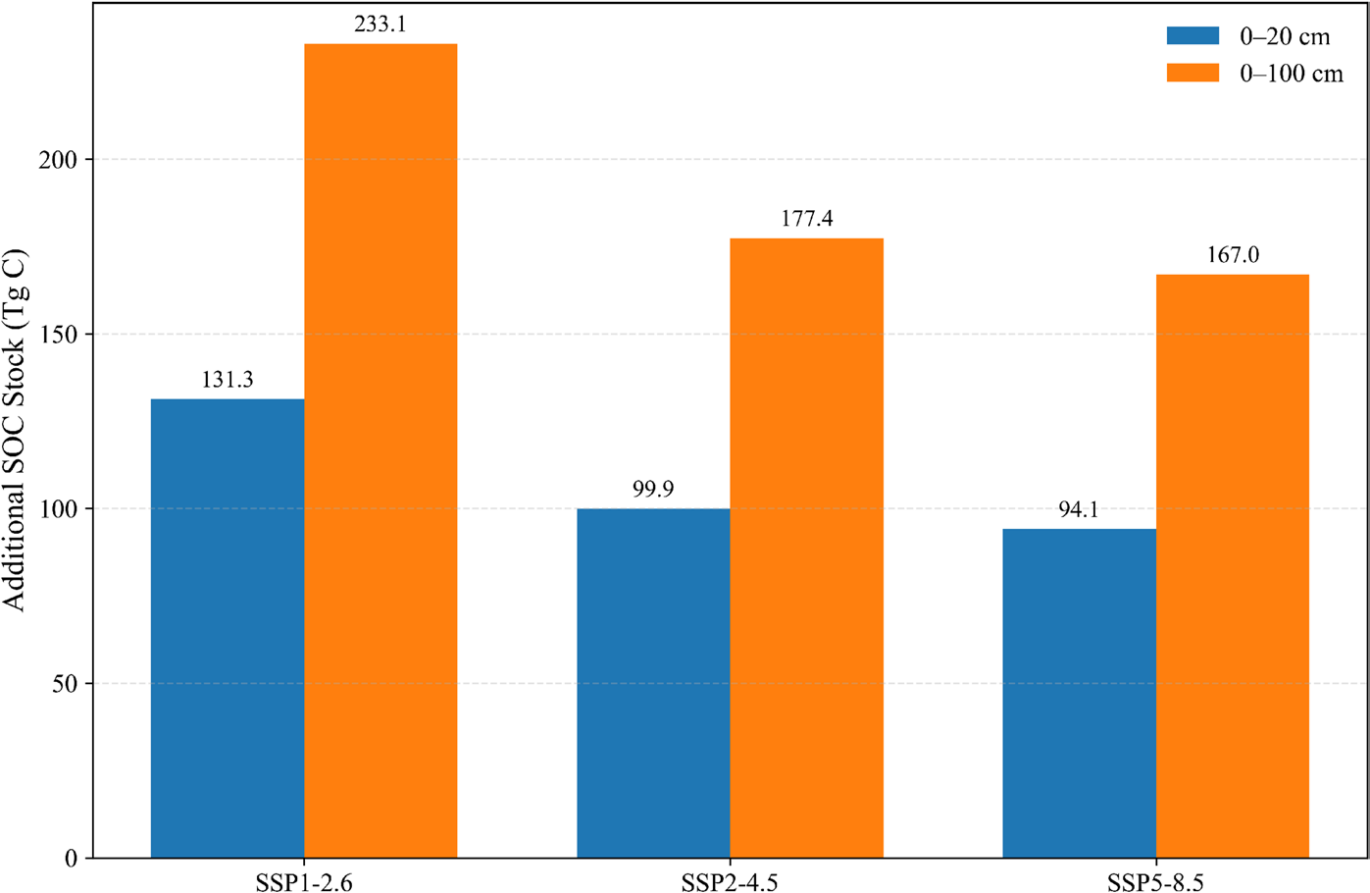
Differences in forest soil organic carbon stocks between protected and unprotected forests under different Shared Socioeconomic Pathways

Protected-area benefits for SOC vary markedly among climate scenarios (Fig. 7). Although protected forests consistently maintain higher SOC stocks than unprotected forests under SSP1-2.6 and SSP2-4.5, this advantage diminishes under SSP5-8.5 as intensified climatic stress increasingly overrides the capacity of protection measures to sustain soil carbon. These results suggest that protected areas serve as effective buffers for SOC conservation, but their effectiveness depends strongly on future climate conditions and is greatest under low- to moderate-emission pathways.

From an applied geographic perspective, these results show that forest SOC is structured jointly by forest composition, soil depth, and spatial governance. Protected areas are consistently associated with higher soil carbon stocks, but their relative contribution depends on both forest type and future climate context. This spatial contingency underscores the importance of integrating soil depth, climate sensitivity, and governance context into conservation prioritization, rather than assuming uniform carbon benefits from protection across all forested territories.

**Fig. 8:**
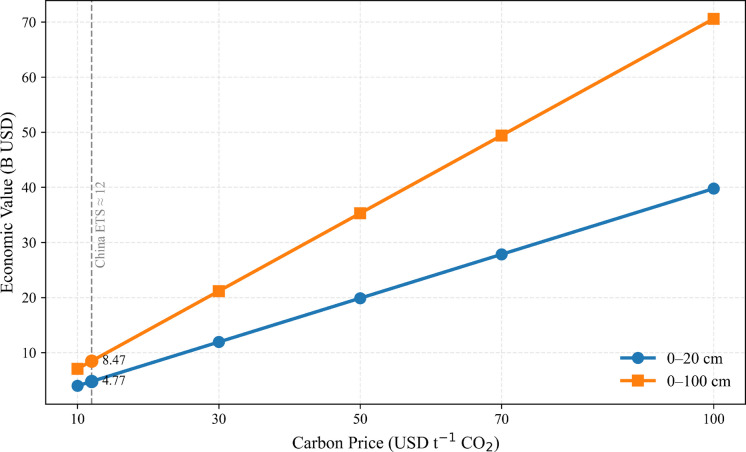
Economic value of the mean increase in soil organic carbon within protected areas under different carbon price scenarios by the end of the twenty-first century, averaged across the SSP1-2.6, SSP2-4.5, and SSP5-8.5 scenarios

When expressed in CO₂-equivalent terms, these additional SOC stocks translate into climate-related economic values that scale strongly with assumed carbon prices (Fig. 8). According to the World Bank, the current price in China’s Emissions Trading System (ETS) is approximately US$12 t^−1^ CO₂. At this price, the mean economic value of the additional soil organic carbon sequestered through protected-area expansion across the three future scenarios is estimated at US$4.77 billion for the 0–20 cm layer and US$8.47 billion for the 0–100 cm layer by the end of the twenty-first century. Across the price range considered, deeper soil layers contribute a larger share of total economic value, reflecting their dominant role in long-term carbon storage. Importantly, the relative ranking of soil depths and protection regimes remains consistent across carbon price scenarios, indicating that spatial contrasts in SOC retention are robust to valuation assumptions.

## Discussion

### Spatial differentiation of forest SOC under climate forcing

This study demonstrates that future forest SOC dynamics in China are strongly spatially differentiated and primarily structured by climatic gradients, with important contrasts between shallow and deep soil layers. Across scenarios, SOC gains are concentrated in high-latitude and colder regions, while widespread losses emerge in subtropical and southern regions under stronger warming. These patterns are consistent with established understanding that lower temperatures suppress decomposition and enhance SOC persistence, whereas warming accelerates microbial activity and destabilizes soil carbon pools (Li et al., 2020; Shi et al., 2024; Zhao et al., 2021).

A key finding is the divergent response between topsoil (0–20 cm) and the full soil profile (0–100 cm). While topsoil SOC may continue to accumulate under certain climate pathways, deeper soil layers exhibit delayed but substantial losses under high-emission scenarios. This depth-dependent sensitivity has been reported in empirical and modelling studies showing that subsoil carbon, despite being more stable in the short term, can incur large cumulative losses once warming thresholds are exceeded (Li et al., 2025a, 2025b; Tian et al., 2016). From an applied geographic perspective, these results highlight the risk of overestimating long-term carbon stability when assessments focus solely on surface soils or vegetation-based indicators.

### Protected areas as spatial governance instruments

Across both soil depths, forests located within protected areas exhibit significantly higher SOC densities than unprotected forests. These differences persist despite substantial variation in climate conditions, forest types, and sample sizes, indicating a consistent spatial association between protection status and soil carbon stocks. Importantly, the results do not imply a direct causal effect of protected-area designation; rather, they reflect long-term territorial contrasts between protected and non-protected landscapes shaped by differences in land-use intensity, disturbance regimes, and management histories.

This interpretation aligns with governance-focused assessments emphasizing that protected areas function as spatial institutions whose ecological outcomes depend on enforcement, land-use restrictions, and broader governance quality (Eklund & Cabeza, 2017; Watson et al., 2018). In China, protected areas often coincide with regions of reduced land conversion, lower fragmentation, and sustained forest cover, conditions that favor SOC retention over decadal timescales (Fan et al., 2023; Tian et al., 2023). The stronger SOC contrasts observed in deeper soil layers further suggest that long-term land-use stability associated with protected areas may contribute to gradual differences in below-ground carbon storage.

### Climate pathways and the limits of governance influence

The magnitude and persistence of SOC differences between protected and non-protected forests are strongly conditioned by future climate pathways. Under low-emission scenarios, forests within protected areas maintain consistently higher SOC stocks across soil depths, whereas under high-emission scenarios this advantage diminishes as climatic constraints increasingly dominate soil carbon dynamics. This finding supports the view that conservation governance can enhance ecosystem functioning where biophysical conditions remain sufficiently flexible but loses leverage under extreme climatic stress (IPBES, 2019; Tian et al., 2023).

From a spatial planning perspective, this conditional effectiveness has important implications. Protected areas are not uniformly effective carbon safeguards across all regions and futures; rather, their relative contribution depends on hydrothermal context and soil depth. Recognizing this spatial contingency is essential for avoiding unrealistic expectations about conservation outcomes under strong climate forcing and for prioritizing regions where governance interventions can still meaningfully influence long-term carbon stability.

### Forest types, territory, and SOC vulnerability

Differences in SOC trajectories among forest types further underscore the importance of geographic context. Coniferous forests, which hold the largest SOC stocks during the historical period, also exhibit the strongest losses under high-emission scenarios. This heightened sensitivity likely reflects their concentration in colder regions where warming exerts a disproportionate effect on decomposition rates (Li et al., 2025a, 2025b; Tian et al., 2016). Mixed forests maintain comparatively stable but much smaller SOC stocks, primarily because they occupy a substantially smaller area than coniferous and broadleaf forests across China.

These results caution against treating forest types as uniform carbon reservoirs. Instead, SOC vulnerability emerges from the interaction between forest composition, climate regime, and soil depth, reinforcing the need for spatially explicit assessments when designing conservation and restoration strategies.

### Climate-related economic value as spatial decision support

By translating SOC stocks into CO₂-equivalent values, this study provides an indicative measure of the climate-related economic value associated with soil carbon retention across territories. The resulting valuations—ranging from tens to over one hundred billion USD depending on soil depth and carbon price—should not be interpreted as market outcomes or policy incentives. Rather, they serve as a spatially comparable metric for illustrating the relative climate-mitigation significance of different regions and governance regimes.

Such valuation approaches are increasingly used to contextualize ecosystem services and inform land-use decisions, particularly when direct market mechanisms are absent or incomplete (Bartkowski et al., 2020; Hungate et al., 2017; Keesstra et al., 2016). In this study, economic value mirrors the spatial and depth-dependent patterns of SOC change, emphasizing that deeper soils contribute disproportionately to long-term mitigation potential. From an applied geography standpoint, this reinforces the argument that below‐ground carbon should be explicitly considered in conservation planning and climate-adaptation strategies, rather than inferred indirectly from above-ground indicators.

### Limitations and future research directions

Several limitations should be acknowledged. First, the analysis focuses on spatial associations rather than causal inference; differences between protected and non-protected forests may partly reflect pre-existing site selection biases rather than direct effects of protection status. Second, future land-use change within and around protected areas is represented indirectly through scenario assumptions rather than explicit policy or behavioral modelling. This limits the ability to isolate governance-driven land-use dynamics under alternative institutional pathways. Third, uncertainty remains regarding long-term responses of deep soil carbon to interacting climate and hydrological changes, particularly under extreme warming scenarios. Although ensemble modeling was used to reduce algorithmic and climate-model uncertainty, deeper soil carbon responses remain inherently difficult to constrain at large spatial scales. Fourth, spatially blocked cross-validation yielded lower performance than random cross-validation, particularly for the 0–100 cm soil layer. This indicates that part of the predictive skill under random splitting was influenced by spatial autocorrelation among nearby samples. While the model retains explanatory power at broader climatic and edaphic gradients, fine-scale spatial generalization, especially for deeper soil carbon pools, remains more uncertain. These findings reinforce that the model is more reliable for national-scale pattern analysis than for local or plot-level prediction.

Future research could integrate finer-scale land-use trajectories, governance effectiveness indicators, and causal inference approaches to better isolate the mechanisms linking protection status and SOC dynamics. For forest-specific applications, incorporating forest-relevant predictors, such as stand age, disturbance history, and canopy structure metrics, alongside dedicated forest soil sampling campaigns would help constrain the unique controls on soil carbon dynamics in forest ecosystems. Extending similar analyses to other regions would also help assess the generality of the spatial patterns observed here.

### Implications for applied geography and conservation planning

Overall, this study highlights that forest SOC is not only a biophysical property but also a spatially governed asset whose distribution and persistence depend on the interaction between climate forcing and territorial management. Protected areas are associated with higher SOC retention where climatic conditions allow governance effects to be expressed, but their effectiveness is uneven across regions and soil depths. By explicitly linking spatial patterns, climate scenarios, and indicative economic value, this research provides an applied geographic framework for identifying where conservation is associated with the greatest long-term climate-mitigation potential.

## Conclusion

This study provides a spatially explicit assessment of how forest SOC and its climate-related value vary across China under future climate change and different governance contexts. The results show that SOC dynamics are strongly structured by climatic gradients and soil depth, with high-latitude forests retaining greater capacity for carbon accumulation and subtropical regions facing heightened vulnerability under warming. Importantly, deeper soil layers exhibit delayed but substantial carbon losses under high-emission scenarios, underscoring the need to consider full soil profiles in long-term climate assessments.

Forests within protected areas consistently exhibit higher SOC densities than unprotected forests across soil depths, reflecting long-term spatial associations linked to reduced land-use intensity and disturbance. However, the magnitude of this association is climate-dependent, indicating that the association between protected-area status and higher SOC retention is climate-dependent.

By translating SOC stocks into CO₂-equivalent terms, this study highlights the often overlooked climate-related value of below‐ground carbon as a territorially differentiated asset. While these valuations are indicative rather than prescriptive, they provide spatially comparable insights that can support conservation prioritization and land-use planning.

Although spatially independent validation indicates reduced predictive performance at fine spatial scales, particularly for deeper soil layers, the model robustly captures broad climatic and edaphic gradients at the national scale. Overall, the findings emphasize that forest SOC is both a biophysical and a spatially governed resource. Integrating soil carbon dynamics into applied geographic analyses can improve the alignment of protected-area planning with long-term climate-mitigation and adaptation objectives.

## Data Availability

Data sets generated during the current study are available from the corresponding author on reasonable request.

## References

[CR1] An, B., Zhang, Z., Xiong, S., Zhang, W., Yi, Y., Liu, Z., & Liu, C. (2024). Landslide susceptibility mapping based on ensemble learning in the Jiuzhaigou region, Sichuan, China. *Remote Sensing,**16*(22), Article 4218. 10.3390/rs16224218

[CR2] Barrena-González, J., Gabourel-Landaverde, V. A., Mora, J., Contador, J. F. L., & Fernández, M. P. (2023). Exploring soil property spatial patterns in a small grazed catchment using machine learning. *Earth Science Informatics,**16*(4), 3811–3838. 10.1007/s12145-023-01125-1

[CR3] Bartkowski, B., Bartke, S., Helming, K., Paul, C., Techen, A.-K., & Hansjürgens, B. (2020). Potential of the economic valuation of soil-based ecosystem services to inform sustainable soil management and policy. *PeerJ,**8*, Article e8749. 10.7717/peerj.8749 32231877 10.7717/peerj.8749 PMC7100588

[CR4] Bócoli, F. A., Ribeiro, D., Mancini, M., de Sousa, L. A., Barbosa, S. M., Serafim, M. E., Silva, B. M., Avanzi, J. C., Guilherme, L. R. G., Curi, N., & Silva, S. H. G. (2025). Can environmental variables, high sampling density and machine learning deliver detailed maps of soil organic carbon and carbon stock in tropical regions? *Catena,**249*, Article 108718. 10.1016/j.catena.2025.108718

[CR5] CGIAR-CSI. (2013). SRTM DEM dataset in China (2000) [Dataset]. National Tibetan Plateau / Third Pole Environment Data Center. https://data.tpdc.ac.cn/zh-hans/data/acb49ce8-2bfe-4ab4-97ff-e6e727110703

[CR6] Chen, J., Biswas, A., Su, H., Cao, J., Hong, S., Wang, H., & Dong, X. (2023). Quantifying changes in soil organic carbon density from 1982 to 2020 in Chinese grasslands using a random forest model. *Frontiers in Plant Science*. 10.3389/fpls.2023.1076902 10.3389/fpls.2023.1076902 PMC10316965 37404537

[CR7] Chen, T., & Guestrin, C. (2016). XGBoost. *Proceedings of the 22nd ACM SIGKDD International Conference on Knowledge Discovery and Data Mining*, 785–794. 10.1145/2939672.2939785

[CR8] Davidson, E. A., & Janssens, I. A. (2006). Temperature sensitivity of soil carbon decomposition and feedbacks to climate change. *Nature,**440*, 165–173. 10.1038/nature04514 16525463 10.1038/nature04514

[CR9] Eklund, J., & Cabeza, M. (2017). Quality of governance and effectiveness of protected areas: Crucial concepts for conservation planning. *Annals of the New York Academy of Sciences,**1399*(1), 27–41. 10.1111/nyas.13284 27918838 10.1111/nyas.13284

[CR10] Eyring, V., Bony, S., Meehl, G. A., Senior, C. A., Stevens, B., Stouffer, R. J., & Taylor, K. E. (2016). Overview of the Coupled Model Intercomparison Project Phase 6 (CMIP6) experimental design and organization. *Geoscientific Model Development,**9*(5), 1937–1958. 10.5194/gmd-9-1937-2016

[CR11] Fan, X., Xu, W., Zang, Z., & Ouyang, Z. (2023). Representativeness of China’s protected areas in conserving its diverse terrestrial ecosystems. *Ecosystem Health and Sustainability*. 10.34133/ehs.0029

[CR12] Fick, S. E., & Hijmans, R. J. (2017). WorldClim 2: New 1‐km spatial resolution climate surfaces for global land areas. *International Journal of Climatology,**37*(12), 4302–4315. 10.1002/joc.5086

[CR13] Griscom, B. W., Adams, J., Ellis, P. W., Houghton, R. A., Lomax, G., Miteva, D. A., Schlesinger, W. H., Shoch, D., Siikamäki, J. V., Smith, P., Woodbury, P., Zganjar, C., Blackman, B., Campari, J., Conant, R. T., Delgado, C., Elias, P., Gopalakrishna, T., Hamsik, M. R., … Fargione, J. (2017). Natural climate solutions. *Proceedings of the National Academy of Sciences of the United States of America,**114*(44), 11645–11650. 10.1073/pnas.1710465114 29078344 10.1073/pnas.1710465114 PMC5676916

[CR14] Hungate, B. A., Barbier, E. B., Ando, A. W., Marks, S. P., Reich, P. B., van Gestel, N., Tilman, D., Knops, J. M. H., Hooper, D. U., Butterfield, B. J., & Cardinale, B. J. (2017). The economic value of grassland species for carbon storage. *Science Advances*. 10.1126/sciadv.1601880 10.1126/sciadv.1601880 PMC5381958 28435876

[CR15] IPBES. (2019). Global assessment report on biodiversity and ecosystem services of the Intergovernmental Science-Policy Platform on Biodiversity and Ecosystem Services . Zenodo. 10.5281/zenodo.6417333

[CR16] Keesstra, S. D., Bouma, J., Wallinga, J., Tittonell, P., Smith, P., Cerdà, A., Montanarella, L., Quinton, J. N., Pachepsky, Y., van der Putten, W. H., Bardgett, R. D., Moolenaar, S., Mol, G., Jansen, B., & Fresco, L. O. (2016). The significance of soils and soil science towards realization of the United Nations Sustainable Development Goals. *SOIL,**2*(2), 111–128. 10.5194/soil-2-111-2016

[CR17] Kottek, M., Grieser, J., Beck, C., Rudolf, B., & Rubel, F. (2006). World map of the Köppen-Geiger climate classification updated. *Meteorologische Zeitschrift (Berlin, Germany : 1992),**15*(3), 259–263. 10.1127/0941-2948/2006/0130

[CR18] Leo, B. (2001). Random forests. *Machine Learning,**45*(1), 5–32.

[CR19] Li, F., Cao, J., Ma, B., Han, F., Geng, J., Zhong, J., Wang, L., & Ma, Y. (2025). Assessing the climate sensitivity of soil organic carbon in China based on machine learning and a bottom-up framework. *Sustainability,**17*(9), Article 3965. 10.3390/su17093965

[CR20] Li, H., Zhou, Y., Zhao, X., Zhang, X., & Liang, S. (2024). A dataset of 0.05-degree leaf area index in China during 1983–2100 based on deep learning network. *Scientific Data, 11*(1), 1122.10.1038/s41597-024-03948-z 10.1038/s41597-024-03948-z PMC11470004 39394222

[CR21] Li, J., Zhang, D., & Liu, M. (2020). Factors controlling the spatial distribution of soil organic carbon in Daxing’anling Mountain. *Scientific Reports,**10*(1), 12659. 10.1038/s41598-020-69590-y 10.1038/s41598-020-69590-y PMC7391772 32728137

[CR22] Li, Y., Wei, X., Yan, J., Du, Z., Lv, Y., & Zhou, H. (2025). Divergent stabilization characteristics of soil organic carbon between topsoil and subsoil under different land use types. *Catena,**252*, Article 108838. 10.1016/j.catena.2025.108838

[CR23] Liu, Y., Li, S., Sun, X., & Yu, X. (2016). Variations of forest soil organic carbon and its influencing factors in east China. *Annals of Forest Science,**73*(2), 501–511. 10.1007/s13595-016-0543-8

[CR24] Loh, W. (2011). Classification and regression trees. *WIREs Data Mining and Knowledge Discovery,**1*(1), 14–23. 10.1002/widm.8

[CR25] Ma, H., & Liang, S. (2022). Development of the GLASS 250-m leaf area index product (version 6) from MODIS data using the bidirectional LSTM deep learning model. *Remote Sensing of Environment,**273*, Article 112985. 10.1016/j.rse.2022.112985

[CR26] Ma, J., Li, J., Wu, W. & Liu, J. (2023). Global forest fragmentation change from 2000 to 2020. *Nature Communications, 14*, 3752. 10.1038/s41467-023-39221-x 10.1038/s41467-023-39221-x PMC10336092 37433782

[CR27] Meersmans, J., De Ridder, F., Canters, F., De Baets, S., & Van Molle, M. (2008). A multiple regression approach to assess the spatial distribution of soil organic carbon (SOC) at the regional scale (Flanders, Belgium). *Geoderma,**143*(1–2), 1–13. 10.1016/j.geoderma.2007.08.025

[CR28] Morais, T. G., Jongen, M., Tufik, C., Rodrigues, N. R., Gama, I., Serrano, J., Gonçalves, M. C., Mano, R., Domingos, T., & Teixeira, R. F. M. (2023). Satellite-based estimation of soil organic carbon in Portuguese grasslands. *Frontiers in Environmental Science*. 10.3389/fenvs.2023.1240106

[CR29] Nwaogu, C., Diagi, B. E., Ekweogu, C. V., Ajeyomi, A. S., Ejiogu, C. C., Emereibeole, E. I., Eneche, P. S. U., Okeke, O. J., Edokpa, D. O., Chike, E., Ozabor, F., Adekunle, O., Wekpe, V. O., Dollah, O. C., Ogaga, E., Nwankwoala, H. O., Wallace, E., Onugu, C., Fajembola, T., & Cherubin, M. R. (2024). Soil organic carbon stocks as driven by land use in Mato Grosso State: The Brazilian Cerrado agricultural frontier. *Discover Sustainability,**5*(1), Article 382. 10.1007/s43621-024-00592-w

[CR30] Peng, S. (2019). *1-km monthly mean temperature dataset for china (1901–2024)*. 10.11888/Meteoro.tpdc.270961

[CR31] Peng, S. (2020). *1-km monthly precipitation dataset for China (1901-2024)*. 10.5281/zenodo.3114194

[CR32] Pierson, D., Lohse, K. A., Wieder, W. R., Patton, N. R., Facer, J., de Graaff, M.-A., Georgiou, K., Seyfried, M. S., Flerchinger, G., & Will, R. (2022). Optimizing process-based models to predict current and future soil organic carbon stocks at high-resolution. *Scientific Reports,**12*(1), 10824. 10.1038/s41598-022-14224-8 35752734 10.1038/s41598-022-14224-8 PMC9233666

[CR33] Pohjankukka, J., Pahikkala, T., Nevalainen, P., & Heikkonen, J. (2017). Estimating the prediction performance of spatial models via spatial k-fold cross validation. *International Journal of Geographical Information Science,**31*(10), 2001–2019. 10.1080/13658816.2017.1346255

[CR34] Qing, Z., Liu, H., Meng, X., Du, B., Zhang, S., & Yu, M. (2025). Assessment of the synergistic effects of future climate change and land use on soil organic carbon stock in Northeast China. *Catena,**260*, Article 109456. 10.1016/j.catena.2025.109456

[CR35] Rao, E., Xiao, Y., Lu, F., Yang, H., & Ouyang, Z. (2023). Preservation of soil organic carbon (SOC) through ecosystems’ soil retention services in China. *Land,**12*(9), Article 1718. 10.3390/land12091718

[CR36] Roberts, D. R., Bahn, V., Ciuti, S., Boyce, M. S., Elith, J., Guillera‐Arroita, G., Hauenstein, S., Lahoz‐Monfort, J. J., Schröder, B., Thuiller, W., Warton, D. I., Wintle, B. A., Hartig, F., & Dormann, C. F. (2017). Cross-validation strategies for data with temporal, spatial, hierarchical, or phylogenetic structure. *Ecography,**40*(8), 913–929. 10.1111/ecog.02881

[CR37] Shi, Z., Hoffman, F. M., Xu, M., Mishra, U., Allison, S. D., Zhou, J., & Randerson, J. T. (2024). Global-scale convergence obscures inconsistencies in soil carbon change predicted by earth system models. *AGU Advances*. 10.1029/2023AV001068

[CR38] Song, B., Wang, M., Zhang, S., Zhang, L., Lu, Y., Guo, H., Guo, X., Zhang, Y., & Zhou, X. (2025). Spatial distribution, drivers, and future variation of soil organic carbon in China’s ecosystems: A meta-analysis and machine-learning assessment. *Ecological Indicators,**179*, Article 114255. 10.1016/j.ecolind.2025.114255

[CR39] Taghizadeh-Mehrjardi, R., Schmidt, K., Amirian-Chakan, A., Rentschler, T., Zeraatpisheh, M., Sarmadian, F., Valavi, R., Davatgar, N., Behrens, T., & Scholten, T. (2020). Improving the spatial prediction of soil organic carbon content in two contrasting climatic regions by stacking machine learning models and rescanning covariate space. *Remote Sensing,**12*(7), Article 1095. 10.3390/rs12071095

[CR40] Tian, J., Feng, C., Fu, G., Fan, L., & Wang, W. (2023). Contribution of different types of terrestrial protected areas to carbon sequestration services in China: 1980–2020. *Frontiers in Ecology and Evolution*. 10.3389/fevo.2023.1074410

[CR41] Tian, Q., He, H., Cheng, W., Bai, Z., Wang, Y., & Zhang, X. (2016). Factors controlling soil organic carbon stability along a temperate forest altitudinal gradient. *Scientific Reports,**6*(1), Article 18783. 10.1038/srep18783 26733344 10.1038/srep18783 PMC4702125

[CR42] van der Westhuizen, S., Heuvelink, G. B. M., & Hofmeyr, D. P. (2023). Multivariate random forest for digital soil mapping. *Geoderma,**431*, Article 116365. 10.1016/j.geoderma.2023.116365

[CR43] Watson, J. E. M., Evans, T., Venter, O., Williams, B., Tulloch, A., Stewart, C., Thompson, I., Ray, J. C., Murray, K., Salazar, A., McAlpine, C., Potapov, P., Walston, J., Robinson, J. G., Painter, M., Wilkie, D., Filardi, C., Laurance, W. F., Houghton, R. A., … Lindenmayer, D. (2018). The exceptional value of intact forest ecosystems. *Nature Ecology & Evolution,**2*(4), 599–610. 10.1038/s41559-018-0490-x 29483681 10.1038/s41559-018-0490-x

[CR44] Xin, Z., Qin, Y., & Yu, X. (2016). Spatial variability in soil organic carbon and its influencing factors in a hilly watershed of the Loess Plateau, China. *Catena,**137*, 660–669. 10.1016/J.CATENA.2015.01.028

[CR45] Xu, L., He, N. P., spsampsps Yu, G. R. (2020). *2010s中国陆地生态系统碳密度数据集* [2010s China Terrestrial Ecosystem Carbon Density Dataset] [DS/OL]. National Ecosystem Science Data Center. 10.11922/sciencedb.603

[CR46] Yang, J., & Huang, X. (2021). The 30 m annual land cover dataset and its dynamics in China from 1990 to 2019. *Earth System Science Data,**13*(8), 3907–3925. 10.5194/essd-13-3907-2021

[CR47] Yang, R. M., Huang, L. M., Zhang, X., Zhu, C. M., & Xu, L. (2023). Mapping the distribution, trends, and drivers of soil organic carbon in China from 1982 to 2019. *Geoderma,**429*, Article 116232. 10.1016/J.GEODERMA.2022.116232

[CR48] Zhang, T., Cheng, C., & Wu, X. (2023). Mapping the spatial heterogeneity of global land use and land cover from 2020 to 2100 at a 1 km resolution. *Scientific Data,**10*(1), Article 748. 10.1038/s41597-023-02637-7 37898602 10.1038/s41597-023-02637-7 PMC10613310

[CR49] Zhao, F., Wu, Y., Hui, J., Sivakumar, B., Meng, X., & Liu, S. (2021). Projected soil organic carbon loss in response to climate warming and soil water content in a loess watershed. *Carbon Balance and Management,**16*(1), Article 24. 10.1186/s13021-021-00187-2 34398330 10.1186/s13021-021-00187-2 PMC8369727

